# 4-Bromo-2-[(*E*)-(2-{2-[(2-{[(*E*)-5-bromo-2-hy­droxy­benzyl­idene]amino}­phen­yl)sulfan­yl]ethyl­sulfan­yl}phen­yl)imino­meth­yl]phenol

**DOI:** 10.1107/S1600536812034071

**Published:** 2012-08-04

**Authors:** Hadi Kargar, Reza Kia, Muhammad Nawaz Tahir

**Affiliations:** a Department of Chemistry, Payame Noor University, PO Box 19395-3697 Tehran, I. R. of IRAN; b Department of Chemistry, Science and Research Branch, Islamic Azad University, Tehran, Iran; c Department of Physics, University of Sargodha, Punjab, Pakistan

## Abstract

The asymmetric unit of the title compound, C_28_H_22_Br_2_N_2_O_2_S_2_, comprises half of a Schiff base ligand, the whole mol­ecule being generated by a crystallographic inversion center located at the mid-point of the C—C bond of the central methyl­ene segment. Intra­molecular O—H⋯N and O—H⋯S hydrogen bonds make *S*(6) and *S*(5) ring motifs, respectively. In the crystal, there are no significant inter­molecular inter­actions.

## Related literature

For standard bond lengths, see: Allen *et al.* (1987 ▶). For hydrogen-bond motifs, see: Bernstein *et al.* (1995 ▶). For background to Schiff base ligands see, for example: Kargar *et al.* (2011 ▶); Kia *et al.* (2010 ▶).
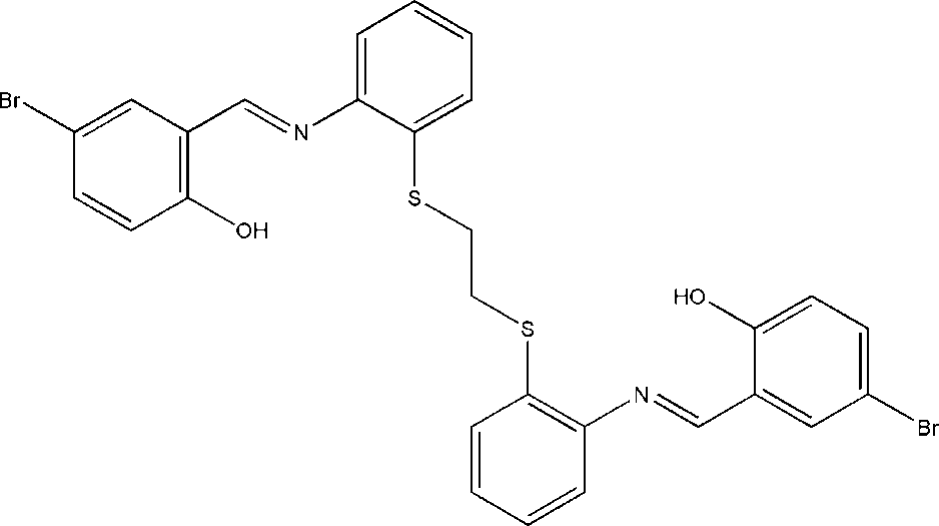


## Experimental

### 

#### Crystal data

C_28_H_22_Br_2_N_2_O_2_S_2_
*M* *_r_* = 642.42
Monoclinic, 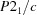

*a* = 13.9124 (18) Å
*b* = 5.4112 (7) Å
*c* = 17.409 (2) Å
β = 92.444 (7)°
*V* = 1309.4 (3) Å^3^
*Z* = 2
Mo *K*α radiation
μ = 3.28 mm^−1^
*T* = 296 K
0.35 × 0.14 × 0.12 mm

#### Data collection

Bruker SMART APEXII CCD area-detector diffractometer
Absorption correction: multi-scan (*SADABS*; Bruker, 2005 ▶) *T*_min_ = 0.393, *T*_max_ = 0.694
10754 measured reflections
2879 independent reflections
1277 reflections with *I* > 2σ(*I*)
*R*_int_ = 0.093

#### Refinement

*R*[*F* ^2^ > 2σ(*F* ^2^)] = 0.052
*wR*(*F* ^2^) = 0.106
*S* = 0.93
2879 reflections
163 parameters
H-atom parameters constrained
Δρ_max_ = 0.38 e Å^−3^
Δρ_min_ = −0.55 e Å^−3^

### 

Data collection: *APEX2* (Bruker, 2005 ▶); cell refinement: *SAINT* (Bruker, 2005 ▶); data reduction: *SAINT*; program(s) used to solve structure: *SHELXS97* (Sheldrick, 2008 ▶); program(s) used to refine structure: *SHELXL97* (Sheldrick, 2008 ▶); molecular graphics: *SHELXTL* (Sheldrick, 2008 ▶); software used to prepare material for publication: *SHELXTL* and *PLATON* (Spek, 2009 ▶).

## Supplementary Material

Crystal structure: contains datablock(s) global, I. DOI: 10.1107/S1600536812034071/su2486sup1.cif

Structure factors: contains datablock(s) I. DOI: 10.1107/S1600536812034071/su2486Isup2.hkl

Supplementary material file. DOI: 10.1107/S1600536812034071/su2486Isup3.cml

Additional supplementary materials:  crystallographic information; 3D view; checkCIF report

## Figures and Tables

**Table 1:** Hydrogen-bond geometry (Å, °)

*D*—H⋯*A*	*D*—H	H⋯*A*	*D*⋯*A*	*D*—H⋯*A*
O1—H1⋯N1	0.90	1.81	2.641 (5)	151
O1—H1⋯S1	0.90	2.74	3.436 (4)	135

**Fig. 1.**
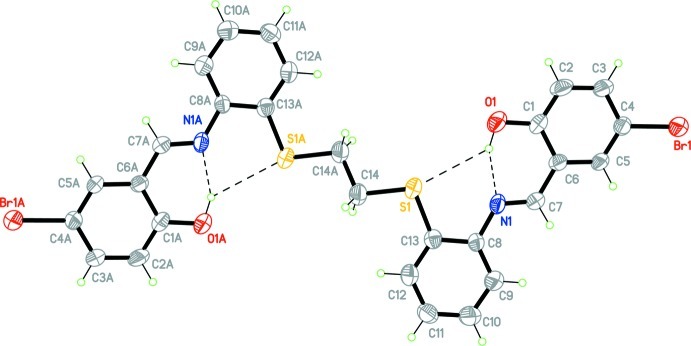
The molecular structure of the title compound, showing 40% probability displacement ellipsoids and the atomic numbering [symmetry code for A: -x, -y + 1, -z + 1]. Dashed lines show the intramolecular interactions

## supplementary crystallographic information

### Comment

In continuation of our work on the crystal structures of Schiff base ligands (Kargar *et al.*, 2011; Kia *et al.*, 2010), we synthesized and determined the X-ray crystal structure of the title compound.

The asymmetric unit of the title compound, Fig. 1, comprises half of a Schiff base ligand. The whole molecule is generated by a crystallographic inversion center located in the middle of the C14—C14^i^ bond of the methylene segment [Symmetry code: (i) -*x*, -*y*+1, -*z*+1]. The bond lengths (Allen *et al.*, 1987) and angles are within the normal ranges. Intramolecular O—H···N and O—H···S hydrogen bonds make *S(6)* and *S(5)* ring motifs, respectively (Table 1; Bernstein *et al.*, 1995).

There are no significant intermolecular interactions in the crystal structure.

### Experimental

The title compound was synthesized by adding 5-bromosalicylaldehyde (2 mmol) to a solution of 2-(2-(2-aminophenylthio)ethylthio)benzenamine (1 mmol) in ethanol (30 ml). The mixture was refluxed with stirring for half an hour. The resultant solution was filtered. Light-yellow needle-like crystals of the title compound, suitable for *X*-ray structure analysis, were obtained by slow evaporation of a solution in ethanol at room temperature over several days.

### Refinement

The O-bound H atom was located in a difference Fourier map and constrained to refine on the parent atom with U_iso_(H) = 1.5U_eq_(O). The C-bound H atoms were included in calculated positions and treated as riding atoms: C-H = 0.93 and 0.97 Å for CH and CH_2_ H atoms, respectively, with U_iso_(H) = 1.2 U_eq_(C).

### Crystal data

**Table:**

C_28_H_22_Br_2_N_2_O_2_S_2_	*F*(000) = 644
*M**_r_* = 642.42	*D*_x_ = 1.629 Mg m^−^^3^
Monoclinic, *P*2_1_/*c*	Mo *K*α radiation, λ = 0.71073 Å
Hall symbol: -P 2ybc	Cell parameters from 2098 reflections
*a* = 13.9124 (18) Å	θ = 2.5–28.8°
*b* = 5.4112 (7) Å	µ = 3.28 mm^−^^1^
*c* = 17.409 (2) Å	*T* = 296 K
β = 92.444 (7)°	Needle, light-yellow
*V* = 1309.4 (3) Å^3^	0.35 × 0.14 × 0.12 mm
*Z* = 2	

### Data collection

**Table:**

Bruker SMART APEXII CCD area-detector diffractometer	2879 independent reflections
Radiation source: fine-focus sealed tube	1277 reflections with *I* > 2σ(*I*)
Graphite monochromator	*R*_int_ = 0.093
φ and ω scans	θ_max_ = 27.1°, θ_min_ = 1.5°
Absorption correction: multi-scan (*SADABS*; Bruker, 2005)	*h* = −17→17
*T*_min_ = 0.393, *T*_max_ = 0.694	*k* = −6→4
10754 measured reflections	*l* = −22→22

### Refinement

**Table:**

Refinement on *F*^2^	Primary atom site location: structure-invariant direct methods
Least-squares matrix: full	Secondary atom site location: difference Fourier map
*R*[*F*^2^ > 2σ(*F*^2^)] = 0.052	Hydrogen site location: inferred from neighbouring sites
*wR*(*F*^2^) = 0.106	H-atom parameters constrained
*S* = 0.93	*w* = 1/[σ^2^(*F*_o_^2^) + (0.0347*P*)^2^] where *P* = (*F*_o_^2^ + 2*F*_c_^2^)/3
2879 reflections	(Δ/σ)_max_ = 0.001
163 parameters	Δρ_max_ = 0.38 e Å^−^^3^
0 restraints	Δρ_min_ = −0.55 e Å^−^^3^

### Special details

**Table:**

Geometry. All esds (except the esd in the dihedral angle between two l.s. planes) are estimated using the full covariance matrix. The cell esds are taken into account individually in the estimation of esds in distances, angles and torsion angles; correlations between esds in cell parameters are only used when they are defined by crystal symmetry. An approximate (isotropic) treatment of cell esds is used for estimating esds involving l.s. planes.
Refinement. Refinement of F^2^ against ALL reflections. The weighted R-factor wR and goodness of fit S are based on F^2^, conventional R-factors R are based on F, with F set to zero for negative F^2^. The threshold expression of F^2^ > 2sigma(F^2^) is used only for calculating R-factors(gt) etc. and is not relevant to the choice of reflections for refinement. R-factors based on F^2^ are statistically about twice as large as those based on F, and R- factors based on ALL data will be even larger.

### Fractional atomic coordinates and isotropic or equivalent isotropic displacement parameters (Å^2^)

**Table:**

	*x*	*y*	*z*	*U*_iso_*/*U*_eq_	
Br1	0.55873 (4)	1.84843 (11)	0.63714 (3)	0.0707 (3)	
S1	0.08346 (11)	0.7463 (2)	0.57293 (8)	0.0622 (4)	
O1	0.2265 (3)	1.2258 (6)	0.51696 (19)	0.0684 (11)	
H1	0.2092	1.1303	0.5566	0.103*	
N1	0.2327 (3)	0.9977 (8)	0.6512 (2)	0.0479 (10)	
C1	0.2994 (4)	1.3675 (9)	0.5463 (3)	0.0502 (13)	
C2	0.3336 (4)	1.5574 (10)	0.5026 (3)	0.0633 (16)	
H2	0.3048	1.5889	0.4544	0.076*	
C3	0.4094 (4)	1.7013 (9)	0.5286 (3)	0.0577 (15)	
H3	0.4322	1.8277	0.4981	0.069*	
C4	0.4522 (3)	1.6562 (9)	0.6015 (3)	0.0492 (13)	
C5	0.4185 (3)	1.4704 (9)	0.6453 (3)	0.0483 (13)	
H5	0.4480	1.4404	0.6934	0.058*	
C6	0.3408 (3)	1.3230 (8)	0.6206 (2)	0.0439 (12)	
C7	0.3043 (4)	1.1325 (9)	0.6696 (3)	0.0510 (13)	
H7	0.3352	1.1076	0.7174	0.061*	
C8	0.1967 (3)	0.8149 (9)	0.7006 (3)	0.0436 (12)	
C9	0.2299 (4)	0.7718 (10)	0.7752 (3)	0.0672 (16)	
H9	0.2797	0.8671	0.7968	0.081*	
C10	0.1892 (4)	0.5876 (10)	0.8175 (3)	0.0692 (17)	
H10	0.2109	0.5615	0.8681	0.083*	
C11	0.1171 (4)	0.4423 (10)	0.7861 (3)	0.0616 (15)	
H11	0.0911	0.3157	0.8148	0.074*	
C12	0.0836 (4)	0.4857 (10)	0.7114 (3)	0.0595 (15)	
H12	0.0338	0.3893	0.6903	0.071*	
C13	0.1228 (3)	0.6697 (9)	0.6678 (3)	0.0447 (12)	
C14	0.0071 (4)	0.4938 (9)	0.5431 (2)	0.0567 (14)	
H14A	0.0368	0.3379	0.5582	0.068*	
H14B	−0.0543	0.5063	0.5671	0.068*	

### Atomic displacement parameters (Å^2^)

**Table:**

	*U*^11^	*U*^22^	*U*^33^	*U*^12^	*U*^13^	*U*^23^
Br1	0.0767 (5)	0.0794 (5)	0.0547 (4)	−0.0273 (3)	−0.0120 (3)	0.0093 (3)
S1	0.0652 (11)	0.0659 (11)	0.0537 (9)	−0.0180 (7)	−0.0170 (7)	−0.0023 (7)
O1	0.059 (3)	0.091 (3)	0.053 (2)	−0.020 (2)	−0.0163 (19)	0.0036 (18)
N1	0.042 (3)	0.054 (3)	0.047 (2)	−0.011 (2)	−0.006 (2)	−0.007 (2)
C1	0.049 (3)	0.059 (4)	0.042 (3)	0.000 (3)	−0.008 (2)	−0.008 (3)
C2	0.060 (4)	0.085 (5)	0.043 (3)	0.002 (3)	−0.014 (3)	0.016 (3)
C3	0.060 (4)	0.070 (4)	0.042 (3)	−0.008 (3)	−0.008 (3)	0.010 (3)
C4	0.048 (3)	0.057 (4)	0.042 (3)	−0.009 (3)	0.001 (2)	−0.003 (3)
C5	0.046 (4)	0.060 (4)	0.038 (3)	−0.007 (3)	−0.009 (2)	0.003 (3)
C6	0.042 (3)	0.052 (4)	0.037 (3)	0.001 (3)	−0.001 (2)	0.003 (3)
C7	0.056 (4)	0.057 (4)	0.039 (3)	−0.007 (3)	−0.006 (3)	0.003 (3)
C8	0.039 (3)	0.047 (4)	0.045 (3)	−0.008 (3)	0.001 (2)	−0.001 (3)
C9	0.064 (4)	0.084 (5)	0.052 (4)	−0.023 (3)	−0.009 (3)	0.007 (3)
C10	0.068 (5)	0.083 (5)	0.056 (4)	−0.011 (3)	−0.002 (3)	0.013 (3)
C11	0.060 (4)	0.061 (4)	0.064 (4)	−0.008 (3)	0.004 (3)	0.013 (3)
C12	0.048 (4)	0.067 (4)	0.062 (4)	−0.014 (3)	−0.007 (3)	0.001 (3)
C13	0.036 (3)	0.047 (3)	0.051 (3)	0.003 (3)	−0.002 (2)	0.000 (3)
C14	0.048 (3)	0.060 (4)	0.062 (3)	−0.009 (3)	−0.003 (3)	−0.005 (3)

### Geometric parameters (Å, º)

**Table:**

Br1—C4	1.894 (5)	C6—C7	1.444 (6)
S1—C13	1.766 (5)	C7—H7	0.9300
S1—C14	1.793 (5)	C8—C9	1.379 (6)
O1—C1	1.354 (5)	C8—C13	1.397 (6)
O1—H1	0.9026	C9—C10	1.376 (6)
N1—C7	1.265 (5)	C9—H9	0.9300
N1—C8	1.416 (5)	C10—C11	1.369 (7)
C1—C2	1.377 (6)	C10—H10	0.9300
C1—C6	1.413 (6)	C11—C12	1.383 (6)
C2—C3	1.373 (6)	C11—H11	0.9300
C2—H2	0.9300	C12—C13	1.379 (6)
C3—C4	1.399 (6)	C12—H12	0.9300
C3—H3	0.9300	C14—C14^i^	1.508 (8)
C4—C5	1.358 (6)	C14—H14A	0.9700
C5—C6	1.396 (6)	C14—H14B	0.9700
C5—H5	0.9300		
			
C13—S1—C14	104.3 (2)	C9—C8—C13	120.1 (5)
C1—O1—H1	104.8	C9—C8—N1	125.2 (5)
C7—N1—C8	123.0 (4)	C13—C8—N1	114.7 (4)
O1—C1—C2	119.0 (4)	C10—C9—C8	119.8 (5)
O1—C1—C6	121.3 (5)	C10—C9—H9	120.1
C2—C1—C6	119.7 (5)	C8—C9—H9	120.1
C3—C2—C1	121.3 (5)	C11—C10—C9	120.9 (5)
C3—C2—H2	119.3	C11—C10—H10	119.5
C1—C2—H2	119.3	C9—C10—H10	119.5
C2—C3—C4	119.4 (5)	C10—C11—C12	119.3 (5)
C2—C3—H3	120.3	C10—C11—H11	120.3
C4—C3—H3	120.3	C12—C11—H11	120.3
C5—C4—C3	119.7 (5)	C13—C12—C11	121.0 (5)
C5—C4—Br1	120.4 (4)	C13—C12—H12	119.5
C3—C4—Br1	119.9 (4)	C11—C12—H12	119.5
C4—C5—C6	122.0 (4)	C12—C13—C8	118.8 (4)
C4—C5—H5	119.0	C12—C13—S1	124.8 (4)
C6—C5—H5	119.0	C8—C13—S1	116.3 (4)
C5—C6—C1	117.7 (4)	C14^i^—C14—S1	107.8 (4)
C5—C6—C7	120.8 (4)	C14^i^—C14—H14A	110.2
C1—C6—C7	121.5 (4)	S1—C14—H14A	110.2
N1—C7—C6	123.7 (5)	C14^i^—C14—H14B	110.2
N1—C7—H7	118.1	S1—C14—H14B	110.2
C6—C7—H7	118.1	H14A—C14—H14B	108.5

Symmetry code: (i) −*x*, −*y*+1, −*z*+1.

### Hydrogen-bond geometry (Å, º)

**Table:**

*D*—H···*A*	*D*—H	H···*A*	*D*···*A*	*D*—H···*A*
O1—H1···N1	0.90	1.81	2.641 (5)	151
O1—H1···S1	0.90	2.74	3.436 (4)	135
